# ﻿Three new wood-inhabiting fungi of *Botryobasidium* (Cantharellales, Basidiomycota) from subtropical forests of Southwestern China

**DOI:** 10.3897/mycokeys.109.133325

**Published:** 2024-10-22

**Authors:** Lin-Jiang Zhou, Xue-Long Li, Hai-Sheng Yuan

**Affiliations:** 1 CAS Key Laboratory of Forest Ecology and Silviculture, Institute of Applied Ecology, Chinese Academy of Sciences, Shenyang 110164, China Institute of Applied Ecology, Chinese Academy of Sciences Shenyang China; 2 University of the Chinese Academy of Sciences, Beijing 100049, China University of the Chinese Academy of Sciences Beijing China; 3 Institute of Edible Fungi, Liaoning Academy of Agricultural Sciences, Shenyang 110161, Liaoning, China Institute of Edible Fungi, Liaoning Academy of Agricultural Sciences Liaoning China

**Keywords:** Botryobasidiaceae, corticioid fungi, subtropical forests, taxonomy, wood-decaying fungi

## Abstract

The basidiomycete genus *Botryobasidium* is a resupinate saprotrophic with a global distribution range from coniferous to broad-leaved forest ecosystems. Though numerous species have been reported from Eurasia and North America, few have been described from China. In the current work, phylogenetic analyses of *Botryobasidium* in China were conducted based on the dataset of the internal transcribed spacer (ITS) regions and the large subunit (LSU) of nuclear ribosomal RNA gene. Maximum likelihood and Bayesian analyses were used to reconstruct the phylogenetic tree, and three new species, namely *Botryobasidiumacanthosporum*, *B.leptocystidiatum* and *B.subovalibasidium*, were described from subtropical forests of Yunnan Province, Southwestern China. *Botryobasidiumacanthosporum* is characterized by having yellowish white to dark yellow basidiome, clavate to tubular cystidia, and subglobose to globose basidiospores with obtuse spines. *Botryobasidiumleptocystidiatum* is characterized by having fluffy to arachnoid, greyish white to ivory basidiome, generative hyphae with clamped, tubular cystidia, and subnavicular to navicular basidiospores. While, *B.subovalibasidium* is characterized by having yellowish to ivory basidiome, subovoid basidia, navicular to suburniform basidiospores, and thick-walled chlamydospores. These three new species are described and illustrated, and the discriminating characters between the new species and their closely related species are discussed. A key to known species of *Botryobasidium* in China is provided.

## ﻿Introduction

*Botryobasidium* Donk belongs to the order Cantharellales of phylum Basidiomycota, and was typified by *B.subcoronatum* (Höhn. & Litsch.) Donk (Moncalvo et al. 2006). Many asexual morph generic names, such as *Acladium* Link, *Allescheriella* Henn., *Alysidium* Kunze, *Haplotrichum* Link, *Neoacladium* P.N. Singh & S.K. Singh, *Physospora* Fr., and *Sporocephalium* Chevall., are congeneric with *Botryobasidium*, and were re-combined in *Botryobasidium* (Stalpers et al. 2021). The genus is characterized by resupinate, smooth, arachnoid, hypochnoid, pellicular or grandinioid basidiomes, a monomitic hyphal system with simple septate or nodose generative hyphae, clavate to cylindrical cystidia, claviform to suburniform basidia with 2–8 sterigmata, and navicular to globose, thin- or thick-walled, smooth or ornamented basidiospores (Binder et al. 2005; Larsson 2007; Buyck et al. 2017; Bondartseva and Zmitrovich 2023). In macromorphology, species of *Botryobasidium* are easily confused with some genera, e.g., *Ceratobasidium* D.P. Rogers, *Sistotrema* Fr., and *Tulasnella* J. Schröt. in the Cantharellales (Donk 1956; Oberwinkler 1982), whereas *Botryobasidium* differs from the others in absence of epibasidia, sturdy and long sterigmata, and oily inclusions (Kotiranta and Saarenoksa 2005; Gorjón and Hallenberg 2008; Oberwinkler et al. 2017). Subsequent molecular phylogenetic studies confirmed the close relationships among *Botryobasidium*, *Cantharellus*, *Clavulina*, *Hydnum* and *Tulasnella* (Jülich 1981; Hibbett et al. 1997; Bruns et al. 1998; Langer 1998; Pine et al. 1999; Cao et al. 2021).

The species of *Botryobasidium* are a group of saprotrophic fungi that cause a white rot in fallen angiosperm and gymnosperm woods, which play a key role in carbon recycling and energy flow in different forest ecosystems (Langer et al. 2000b; Bondartseva and Zmitrovich 2023). They can be commonly found on various hosts or substrates from the litter, fallen trunk to stem of living trees, including the macrophanerophytes, such as *Abies* Mill, *Acer* Linn., *Alnus* Mill., *Betula* L., *Citrus* L., *Corylus* L., *Eucalyptus* L. Herit, *Fagus* L., *Magnolia* Linn., *Persea* Mill., *Picea* Dietr., *Pinus* Linn, *Populus* L., *Quercus* L., *Salix* L. and *Tsuga* Carr.; the shrubs, such as *Bambusa* Retz. corr. Schreber and *Pandanus* Linn. f.; the pteridophytes, such as *Pteris* L. and *Cibotium* Kaulf. (Anon 1969; Holubová-Jechová 1969; Boidin and Gilles 1982; Langer 1994; Langer et al. 2000a, 2000b; Hjortstam et al. 2005). Additionally, some species also develop on mature basidiomes of *Irpexlacteus* (Boidin and Gilles 1982), as well as on soil and underground timber (Anon 1969).

Up to now, the genus of about 84 species have been accepted globally in Index Fungorum and MycoBank (Lentz 1967; Jung 1995; Langer et al. 2000a; Hagara 2001; Bernicchia et al. 2010; Saitta et al. 2011; Bates et al. 2017; Kalinina et al. 2020; Ram et al. 2021). There have been recorded about 35 species in Europe, 28 in North America, 26 in Latin America, 25 in Africa, 20 in Oceania, and 23 in Asia (Dritter 1809; Anon 1969; Holubová-Jechová 1969; Pouzar and Holubová-Jechová 1969; Holubová-Jechová 1980; Boidin and Gilles 1982; Boidin and Gilles 1988; Langer 1994; Greslebin and Rajchenberg 2003; Parmasto et al. 2004; Hjortstam et al. 2005; Bates et al. 2017; Buyck et al. 2017; Hyde et al. 2019; Vondrák et al. 2023). So far, 15 species of *Botryobasidium* have been reported from China, and most of them were distributed in the north temperate to subtropical zones (Dai 2011; Liu et al. 2024; Zhou et al. 2024).

During the surveys of lignicolous fungi in Yunnan Province, Southwestern China, several *Botryobasidium* specimens were collected from the mixed forests. The subsequent research by morphology and molecular phylogeny indicates that these specimens represent several undescribed species. The phylogenetic positions and the relationships of these species among *Botryobasidium* were clarified based on ITS + LSU dataset, and descriptions of these species with line drawings were provided in this study.

## ﻿Materials and methods

### ﻿Morphological study

The voucher specimens are deposited in the herbarium of the Institute of Applied Ecology, Chinese Academy of Sciences (IFP). Macromorphological characteristics were examined using a Nikon SMZ 645 (Tokyo, Japan) stereo microscope and the color descriptions refer to Kornerup and Wanscher (1981). Microscopical structures were checked using hand-cut sections stained with Cotton blue, Melzer’s reagent, and 3% KOH, and line drawings were prepared using a Nikon Eclipse 80i microscope (Nikon Corporation, Japan) with the aid of a drawing tube. The surface morphology for the basidiospores was observed with a field emission scanning electron microscope (SEM5000S, CIQTEK Co., Ltd.) at an accelerating voltage of 3 kV. The working distance was 9.62 mm. A thin layer of gold was plated on the sample to enhance the conductivity. Basidiospores were measured based on the front and back side view; the apex was excluded from the spore measurements. The following abbreviations are used: L = mean spore length, W = mean spore width, Q = L/W ratio, n (a/b) = number of spores (a) measured from number of specimens (b). Cotton blue (CB) was employed as a fitting medium to identify cyanophilous. Potassium hydroxide solution (KOH) was used to detect changes in hyphae, gloeocystidia, and encrusted. Melzer’s reagent (IKI) was used to determine amyloidity and dextrinoidity.

### ﻿DNA extraction, PCR amplification, and DNA sequencing

According to the manufacturer’s instructions, the Fungal Fast Non-Toxic DNA Extraction Kit (Demeter Biotech Co., Ltd, Beijing, China) was employed to extract the sample’s total DNA and amplified by the polymerase chain reaction (PCR). The internal transcribed spacer (ITS) regions were amplified with the primers ITS1 and ITS4 (White et al. 1990), and the procedure was an initial denaturation at 95 °C for 3 min, followed by 34 cycles at 95 °C for 30 s, 58 °C for 30 s, and 72 °C for 1 min, with a final extension at 72 °C for 5 min. The large subunit of nuclear ribosomal RNA gene (LSU) was amplified with the primers LR0R and LR7 (Vilgalys and Hester 1990), and the procedure involved an initial denaturation at 95 °C for 3 min, followed by 34 cycles at 95 °C for 30 s, 50 °C for 30 s, and 72 °C for 1 min, the procedure ended with an extension at 72 °C for 5 min.

DNA sequencing was conducted at the Beijing Genomics Institute (BGI), and the sequences were assembled using Geneious v.9.0.2 (Kearse et al. 2012). The generated sequences were verified and controlled to ensure their quality and integrity, and uploaded to GenBank (Table 1).

**Table 1.** Species and GenBank numbers used in phylogenetic analysis in this study.

Species name	ITS	LSU	Specimen No.	Substrate	Country	References
*Botryobasidiumacanthosporum* L.J. Zhou & H.S. Yuan	PP229497	/	Yuan16326	on fallen angiosperm branch	China	Present study
*B.acanthosporum*	PP229511	/	Yuan17989	on bark of angiosperm	China	Present study
*B.acanthosporum*	PP229512	PP218361	Yuan18083	on fallen trunk of *Abies*	China	Present study
*B.acanthosporum*	PP229517	/	Yuan18128	on fallen trunk of *Abies*	China	Present study
*Botryobasidiumaureum* Parmasto	AJ389783	/	GEL 2910	/	Germany	Langer et al. 2000b
*B.botryosum* (Bres.) J. Erikss.	DQ267124	DQ089013	AFTOL-ID 604	/	USA	AFTOL Database
*B.candicans* J. Erikss.	KP814200	/	UC2022893	on litter or well decayed wood in pinaceous forest	USA	Rosenthal et al. 2017
B.cf.subcoronatum	KP814216	/	UC2022856	on litter or well decayed wood in pinaceous forest	USA	Rosenthal et al. 2017
B.cf.subcoronatum	KP814322	/	UC2022917	on litter or well decayed wood in pinaceous forest	USA	Rosenthal et al. 2017
*B.coniferarum* S.L. Liu & L.W. Zhou	PP209210	PP218367	Yuan18440	on fallen gymnosperm trunk	China	Present study
*B.coniferarum*	OR557262	OR527286	LWZ20171016-15	on fallen branch of *Pinus*	*China*	Liu et al. 2024
*B.coniferarum*	OR557259	OR527282	LWZ20210928-3	on fallen branch of *Pinus*	*China*	Liu et al. 2024
*B.conspersum* J. Erikss.	DQ911612	DQ521414	PBM 2747 (CUW)	/	USA	AFTOL Database
*B.conspersum*	OP163274	/	FLAS-F-69114	/	USA	NCBI Database
*B.conspersum*	/	AY586657	GB/KHL11063	/	Sweden	Larsson et al. 2004
*B.curtisii* (Berk.) Hol.-Jech.	EU118629	EU118629	KHL 12950GB	/	Costa Rica	Larsson 2007
*B.gossypirubiginosum* Q. Zhou & C.L. Zhao	OR668924	OR708665	CLZhao 26052	on fallen angiosperm branch	China	Zhou et al. 2024
*B.incanum* Q. Zhou & C.L. Zhao	OR668923	OR708664	CLZhao 26697	on fallen angiosperm branch	China	Zhou et al. 2024
*B.incanum*	PP209201	PP218357	Yuan17803	on fallen angiosperm branch	China	Present study
*B.indicum* (P.N. Singh & S.K. Singh) R. Kirschner & G. Langer	PP209209	PP218363	Yuan18250	on root of *Quercus*	China	Present study
*B.indicum*	ON406471	/	CLZhao 21791	/	China	NCBI Database
*B.indicum*	NR171230	NG070816	AMH:10054	dead bark of *Leucaenaleucocephala*	India	Hyde et al. 2019
*B.indicum*	MK391496	MK391493	AMH:10054	dead bark of *Leucaenaleucocephala*	India	Hyde et al. 2019
*B.intertextum* (Schwein.) Jülich & Stalpers	KP814540	/	UC2022959	on litter or well decayed wood in pinaceous forest	USA	Rosenthal et al. 2017
*B.intertextum*	AJ389782	/	DAOM 197881	/	Canada	Langer et al. 2000b
*B.isabellinum* (Fr.) D.P. Rogers	MZ159478	/	K(M):181602	/	UK	NCBI Database
*B.leptocystidiatum* L.J. Zhou & H.S. Yuan	PP209211	PP218178	Yuan17548	on fallen branch of *Pinus*	China	Present study
*B.leptocystidiatum*	PP204173	PP218180	Yuan17557	on dead tree of *Pinus*	China	Present study
*B.leptocystidiatum*	PP209200	PP218353	Yuan17706	on fallen angiosperm trunk	China	Present study
*B.leptocystidiatum*	PP209197	PP218354	Yuan17708	on bark of living angiosperm tree	China	Present study
*B.leptocystidiatum*	PP209198	PP218355	Yuan17709	on fallen angiosperm trunk	China	Present study
*B.robustius* Pouzar & Hol.-Jech.	MH859491	MH871272	CBS:945.69	/	Czech	Vu et al. 2019
*B.robustius*	PP436446	/	HAY-F-004374	/	USA	NCBI Database
*B.subcoronatum* (Höhn. & Litsch.) Donk	EU118607	EU118607	KHL s.n. (GB)	/	Sweden	Larsson 2007
*B.subcoronatum*	MH211720		FLAS-F-61064	/	USA	NCBI Database
*B.subcoronatum*	DQ200924	AY647212	AFTOL-ID 614	/	USA	Matheny et al. 2007
*B.subovalibasidium*. L.J. Zhou & H.S. Yuan	PP209199	PP218152	Yuan16439	on fallen trunk of *Hippophaerhamnoides*	China	Present study
*B.subovalibasidium*	PP209196	PP218362	Yuan18179	on fallen trunk of *Abies*	China	Present study
*B.tubulicystidium* G. Langer	OL436769	/	DK14_139	/	USA	NCBI Database
*B.vagum* (Berk. & M.A. Curtis) D.P. Rogers	OR680661	/	personal:Alden Dirks:ACD0672	/	USA	Zhou et al. 2024
*B.vagum*	OR471092	/	TENN:075258	on *Pinus*	USA	Zhou et al. 2024
*B.yunnanense* Q. Zhou & C.L. Zhao	OR668925	OR708666	CLZhao 24877	on fallen angiosperm branch	China	Zhou et al. 2024
*Suillosporiumcystidiatum* (D.P. Rogers) Pouzar	MN937573	MN937573	VS3830	On Piceajezoensisvar.jezoensis	Russia	NCBI Database

### ﻿Phylogenetic analyses

*Suillosporiumcystidiatum* (D.P. Rogers) Pouzar (Botryobasidiaceae) was chosen as the outgroup according to the result of sequence BLAST in NCBI database, ensuring that it has suitable phylogenetic distances from other species in *Botryobasidium*. The concatenated datasets of ITS and LSU sequences of the species in Botryobasidiaceae were used to infer the molecular phylogeny. The ITS and LSU sequences were aligned separately using MEGA v.7.0 (Kumar et al. 2016). Maximum likelihood (ML) analysis was done using RAxML v.1.5b2 (Silvestro and Michalak 2012) with non-parametric bootstrapping of 500 replicates under the GTRGAMMA model. A Bayesian inference (BI) was also performed for the same data sets using MrBayes 3.2.6 (Ronquist et al. 2012). A substitution model was selected in PhyloSuite v1.2.2 (Zhang et al. 2020). The Bayesian information criterion (BIC) values under each model were compared and the model with the lowest BIC value was selected. Two parallel analyses were then run in MrBayes for 2 million generations, with 4 chains each, sampling every 500 generations. Burn-in trees (initial 25%) were discarded for each run and posterior probabilities of the matrix were determined by calculating a majority-rule consensus tree generated from the post-burnin trees by the MCMC runs using the sump of MrBayes. The phylogenetic trees were visualized using FigTree v1.4.3 (Rambaut 2016). Branches that received bootstrap support for ML (ML-BS) ≥ 70% and BI (BPP) ≥ 0.95 were considered signiﬁcantly supported, respectively. The datasets were deposited in TreeBASE (www.treebase.org/treebase-web/, study no. 31569).

## ﻿Results

### ﻿Phylogeny

The ITS dataset consists of 39 sequences representing 20 taxa of *Botryobasidium*, and a sample of *Suillosporiumcystidiatum* as the outgroup. The ITS sequence had an aligned length of 661 base pairs (bp), of which 321 were parsimony-informative, 75 were singleton sites, 265 were constant sites. The Bayesian analysis had an average standard deviation of split frequencies = 0.004148, and a 50% majority-rule consensus phylogram was generated. The best model was GTR + F + G4 [lset nst = 6, rates = Gamma, Ngammacat = 4, prset statefreqpr = dirichlet (1, 1, 1, 1)]. The ITS + LSU dataset consists of 40 sequences representing 20 taxa of *Botryobasidium*, and a sample of *Suillosporiumcystidiatum* as the outgroup. The ITS + LSU dataset had an aligned length of 1502 bp (including 663 bp of ITS and 839 bp of LSU), of which 434 were parsimony-informative, 158 were singleton sites, 910 were constant sites. The Bayesian analysis had an average standard deviation of split frequencies = 0.005929, and a 50% majority-rule consensus phylogram was generated. The best model was GTR + F + I + G4 [lset nst = 6, rates = invgamma, Ngammacat = 4, prset statefreqpr = dirichlet (1, 1, 1, 1)].

**Figure 1.**
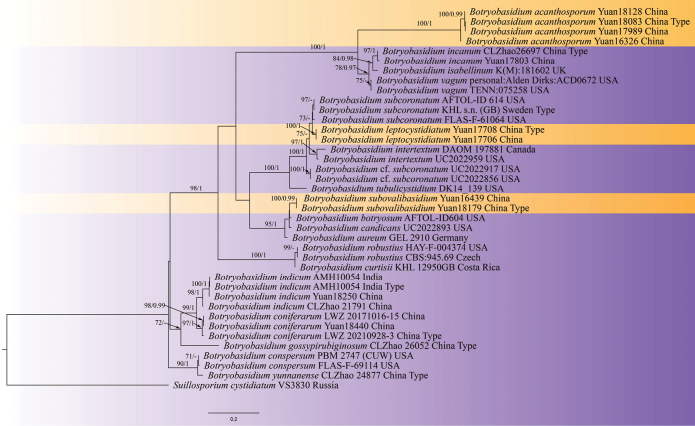
Phylogram of *Botryobasidium* resulting from a maximum likelihood analysis based on ITS sequence. Maximum likelihood bootstrap values (ML, ≥ 70%; left) and Bayesian posterior probabilities (BPP, ≥ 0.95; right) are given at the nodes. New species are in yellow background.

In the phylogenetic tree based on ITS dataset (Fig. 1), four specimens of *B.acanthosporum* formed a clade (ML 100%/BPP 1), and grouped with *B.incanum*, *B.vagum*, and *B.isabellinum* with full support (ML 100%/BPP 1). Two specimens of *B.leptocystidiatum* formed a clade with full support (ML 100%/BPP 1). The remaining two specimens of *B.subovalibasidium* formed a clade, and clustered with *B.aureum*, *B.botryosum* and *B.candicans* with strong support (ML 95%/BPP 1).

**Figure 2.**
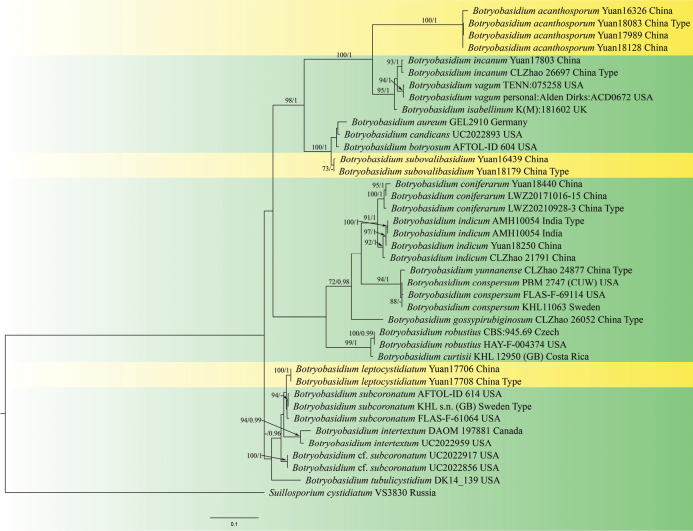
Phylogram of *Botryobasidium* resulting from a maximum likelihood analysis based on ITS + LSU. Maximum likelihood bootstrap values (ML, ≥ 70%; left) and Bayesian posterior probabilities (BPP, ≥ 0.95; right) are given at the nodes. New species are in yellow background.

In the phylogenetic trees based on ITS + LSU dataset (Fig. 2), the branches to which two new species belong swapped positions, but the taxonomic positions of these three new species and the relationships with their sibling species are no discrepancy. Moreover, the support of the branches to which two new species belong, *B.acanthosporum* and *B.subovalibasidium*, was strengthened (ML 98%/BPP 1). Thus, the phylogenetic analyses revealed the taxonomic positions of these three new species.

### ﻿Taxonomy

#### ﻿ Botryobasidium acanthosporum

Taxon classificationFungiCantharellalesBotryobasidiaceae

L.J. Zhou & H.S. Yuan sp. nov.

6DFEAACF-5B38-5AFC-817D-2A02BBC84131

Fungal Names: FN 572031

Figs 3A, B , 4 , 5

##### Diagnosis.

Differed from other *Botryobasidium* species in having arachnoid basidiome with attached granules, clavate to subcylindrical cystidia, and subglobose to globose basidiospores with blunt spines up to 4 µm long.

##### Type.

China • Yunnan Province, Diqing Prefecture, Pudacuo National Park, 27°53'54"N, 99°57'04"E, on fallen trunk of *Abies*, 14 August 2023, *Yuan 18083* (IFP 19972).

##### Etymology.

*acanthosporum* (Lat.), referring to the spore with spines.

##### Description.

***Basidiomes***: annual, adnate and resupinate, fluffy, pellicular, arachnoid with attached granules, 50–150 μm thick, adherent to the substrate and separates easily when wet. Hymenophoral surface smooth, greyish white to yellowish white (1B2–4B2) when fresh, pale yellow to dark yellow (3A3–4C8) when dry. Sterile margin often indeterminate and not differentiated.

**Figure 3.**
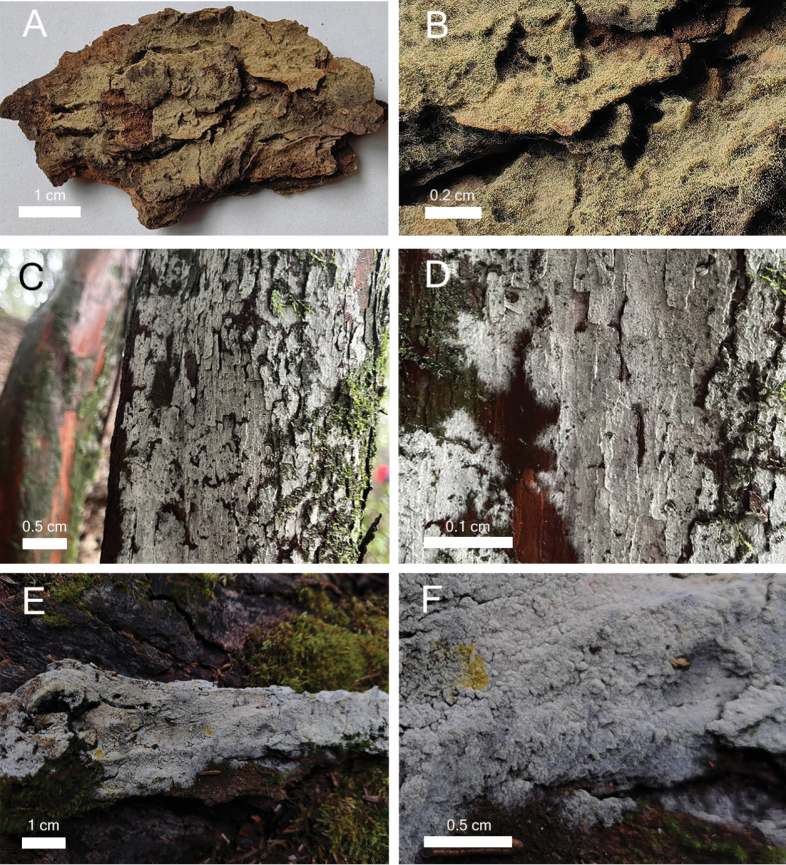
The habitats and basidiomes of three new species of *Botryobasidium***A, B***B.acanthosporum* (holotype Yuan 18083) **C, D***B.leptocystidiatum* (holotype Yuan 17708) **E, F***B.subovalibasidium* (holotype Yuan 18179).

***Hyphal structure***: hyphal system monomitic; generative hyphae simple septate, thin- to slightly thick-walled; tissues unchanged in KOH.

***Subiculum***: subicular hyphae colorless, thick-walled, frequently branched at right angles, cyanophilous, inamyloid, loosely interwoven, 7–12 μm in diam. Subhymenial hyphae colorless, thin-walled, acyanophilous, inamyloid, 7–11 μm in diam.

**Figure 4.**
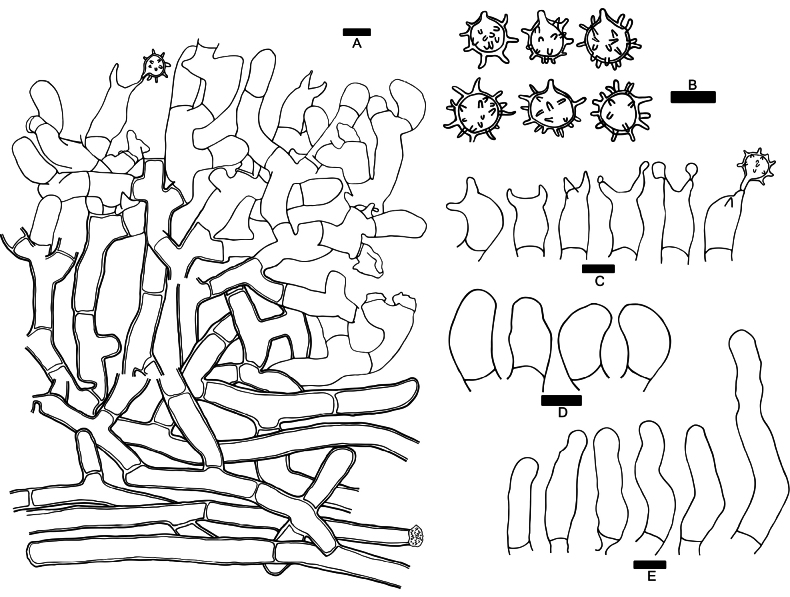
Microscopic features of *Botryobasidiumacanthosporum* (drawn from holotype Yuan 18083) **A** a section through basidiome **B** basidiospores **C** basidia **D** basidioles **E** leptocystidia. Scale bars: 10 μm.

***Cystidia***: clavate to tubular, infrequent, smooth, thin-walled, colorless, simple septate, apically obtuse, acyanophilous, inamyloid, unchanged in KOH and distilled water, 26–37(–64) × 7–10 μm.

***Basidia***: clavate to subcylindrical, smooth, thin-walled, with 2 sterigmata, simple septate, acyanophilous, inamyloid, unchanged in KOH and distilled water, 14.5–20 × 8–10 μm.

**Figure 5.**
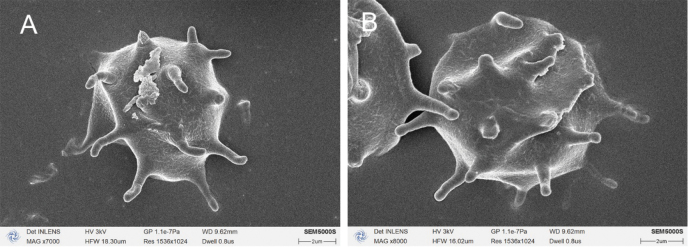
SEM of basidiospores of *Botryobasidiumacanthosporum* species (holotype Yuan 18083).

***Basidiospores***: subglobose to globose, aculeate, slightly thick- to thick-walled, colorless, cyanophilous, inamyloid, unchanged in KOH and distilled water, 8–10(–10.3) × 8–10 μm (exclude spines), L = 9.25 μm, W = 8.92 μm, Q = 1.0–1.13 (n = 60/2); spines with apically obtuse, usually isolated, sometimes grouped in 2, up to 4 µm long.

Chlamydospores absent and anamorph not seen.

##### Ecology and distribution.

Growing in mixed forests dominated by *Abies* and a small number of *Picea*, *Quercus*, and other angiosperm trees. So far, known from Yunnan Province and Xizang Autonomous Region, China.

##### Additional specimens examined.

China • Xizang Autonomous Region, Bomi County, Yigong Tea Farm, 30°07'55"N, 95°01'05"E, on fallen angiosperm branch, 24 October 2021, *Yuan 16326* (IFP 19970; paratype) • Yunnan Province, Diqing Prefecture, Baimaxueshan National Nature Reserve, 28°18'19"N, 99°08'57"E, on bark of angiosperm, 13 August 2023, *Yuan 17989* (IFP 19971) • Pudacuo National Park, 27°53'56"N, 99°57'16"E, on fallen trunk of *Abies*, 14 August 2023, *Yuan 18128* (holotype IFP 19973).

#### ﻿ Botryobasidium leptocystidiatum

Taxon classificationFungiCantharellalesBotryobasidiaceae

L.J. Zhou & H.S. Yuan sp. nov.

CB91A294-5EE4-554F-A6B8-0A8E56DB662C

Fungal Names: FN 571970

Figs 3C, D , 6

##### Diagnosis.

Differed from other *Botryobasidium* species in having tubular cystidia and clamped in all hyphae.

##### Type.

China • Yunnan Province, Lincang City, Wulaoshan National Forest Park, 23°54'47"N, 100°10'53"E, on bark of living angiosperm tree, 9 August 2023, *Yuan 17708* (holotype IFP 019955).

##### Etymology.

*leptocystidiatum* (Lat.), referring to the leptocystidia.

##### Description.

***Basidiomes***: annual, resupinate, effuse, pellicular, fluffy to arachnoid, 100–150 μm thick, adherent to the substrate and not easily separated. Hymenophoral surface smooth, greyish white (1B1–30B1) to smoky grey (3C2) when fresh, greyish white (1B1–30B1) to ivory (4B3) when dry; margin often indeterminate and not differentiated.

***Hyphal structure***: hyphal system monomitic; generative hyphae clamped, thin- to slightly thick-walled; tissues unchanged in KOH.

***Subiculum***: subicular hyphae colorless, slightly thick-walled, sparsely branched at right angles, cyanophilous, inamyloid, loosely interwoven, 7–10 μm in diam. Subhymenial hyphae colorless, thin-walled, frequently branched at right angles, acyanophilous, inamyloid, loosely interwoven, 4–7 μm in diam.

**Figure 6.**
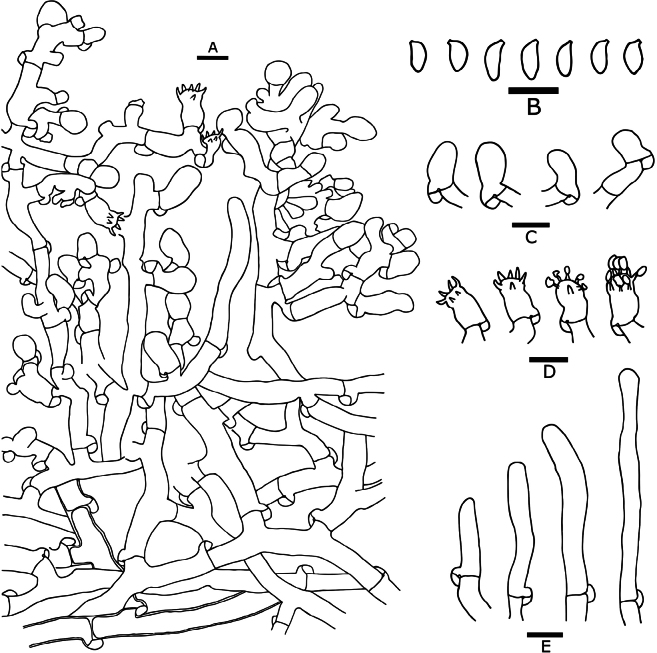
Microscopic features of *Botryobasidiumleptocystidiatum* (drawn from holotype Yuan 17708) **A** a section through basidiome **B** basidiospores **C** basidioles **D** basidia **E** leptocystidia. Scale bars: 10 μm.

***Cystidia***: tubular, infrequent, smooth, thin-walled, colorless, apically obtuse, basal clamped, without additional septate, acyanophilous, inamyloid, unchanged in KOH and distilled water, 21.5–77 × 4–7.5 μm.

***Basidia***: ordered by botryose cluster, subcylindrical, smooth, thin-walled, usually with 6 sterigmata, occasionally with 7 sterigmata, basal clamped, acyanophilous, inamyloid, unchanged in KOH and distilled water, 10.5–15 × 7–8 μm.

***Basidiospores***: subnavicular to navicular, smooth, thin-walled, colorless, occasionally a few stuck together, acyanophilous, inamyloid, unchanged in KOH and distilled water, (6–)6.5–7.8(–8.1) × (2.8–)2.9–3.7(–3.9) μm, L = 7.2 μm, W = 3.1 μm, Q = 1.92–2.5 (n = 120/3).

Chlamydospores absent and anamorph not seen.

##### Ecology and distribution.

Growing in mixed forests dominated by *Pinus* and a small number of Fagaceae trees. So far only known from Yunnan Province, China.

##### Additional specimens examined.

China • Yunnan Province, Lincang City, Wulaoshan National Forest Park, 23°54'47"N, 100°10'53"E, on fallen branch of *Pinus*, 8 August 2023, *Yuan 17548* (IFP 019952; paratype) • on dead tree of *Pinus*, 8 August 2023, *Yuan 17557* (IFP 019953) • on fallen angiosperm trunk, 9 August 2023, *Yuan 17706* (IFP 019954), *Yuan 17709* (IFP 019956).

#### ﻿ Botryobasidium subovalibasidium

Taxon classificationFungiCantharellalesBotryobasidiaceae

L.J. Zhou & H.S. Yuan sp. nov.

4FB91763-7382-51E3-843A-92D77BEA14CE

Fungal Names: FN 571974

Figs 3E, F , 7

##### Diagnosis.

Differed from other *Botryobasidium* species in having effuse, yellowish to ivory basidiomes, subovoid to ovoid basidia, ellipsoid chlamydospores.

##### Type.

China • Yunnan Province, Diqing Prefecture, Pudacuo National Park, 27°83'67"N, 99°95'76"E, Alt. 3655 m, on fallen trunk of *Abies*, 15 August 2023, *Yuan 18179* (holotype IFP 019957).

##### Etymology.

*subovalibasidium* (Lat.), referring to the subovoid basidia.

##### Description.

***Basidiomes***: annual, resupinate, effuse, fluffy, 100–200 µm thick, adherent to the substrate and not easily separated. Hymenophoral surface smooth, greyish white (1B1–30B1) to ivory (4B3) when fresh, pale yellow (4A3) to greyish yellow (4B5) when dry; margin not differentiated, distinct.

***Hyphal structure***: hyphal system monomitic; generative hyphae simple septate, thin- to slightly thick-walled; tissues unchanged in KOH.

***Subiculum***: subicular hyphae colorless, slightly thick-walled, frequently branched, cyanophilous, inamyloid, loosely interwoven, (7–)8–11.5 μm in diam. Subhymenial hyphae colorless, thin-walled, moderately branched, acyanophilous, inamyloid, loosely interwoven, 5–8.5 μm in diam.

**Figure 7.**
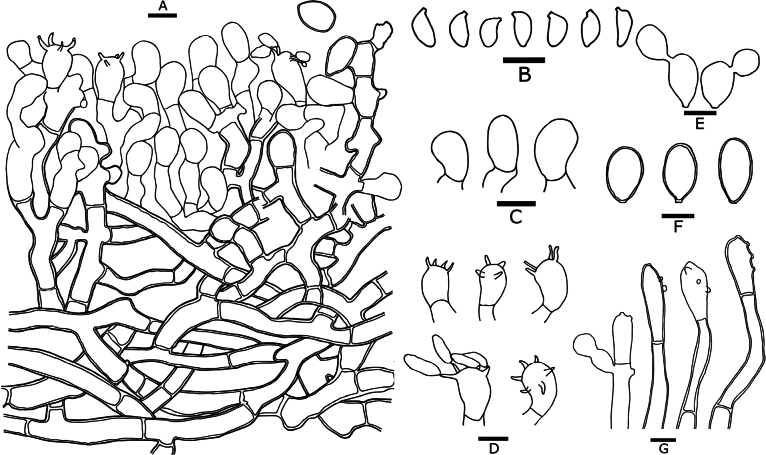
Microscopic features of *Botryobasidiumsubovalibasidium* (drawn from holotype Yuan 18179) **A** a section through basidiome **B** basidiospores **C** basidioles **D** basidia **E** secondary spores **F** chlamydospores **G** conidiophores. Scale bars: 10 μm.

***Cystidia***: absent.

***Basidia***: subovoid to ovoid, smooth, thin-walled, with 4–6 sterigmata, basal simple septate, acyanophilous, inamyloid, unchanged in KOH and distilled water, (12–)14–18 × 9–10 µm.

***Basidiospores***: navicular to suburniform, smooth, thin-walled, colorless, occasionally stuck together, acyanophilous, inamyloid, unchanged in KOH and distilled water, (5.7–)7–9.8(–10) × (3.2–)3.7–5(–5.1) µm, L = 8.3 µm, W = 4.2 µm, Q = 1.53–2.5 (n = 60/1).

***Chlamydospores***: orange-yellow, ellipsoid, smooth, thick-walled, cyanophilous, inamyloid, unchanged in KOH, unchanged in distilled water, 17–21(–22) × (9–)10–11 µm, L = 18.5 µm, W = 10.3 µm, Q = 1.50–2.1 (n = 60/2).

##### Ecology and distribution.

Growing in mixed forests dominated by *Abies* and a small number of *Picea*, *Quercus*, and other angiosperm trees. So far, known from Yunnan Province and Xizang Autonomous Region, China.

##### Additional specimen examined.

China • Xizang Autonomous Region, Bomi County, on fallen trunk of *Hippophaerhamnoides*, 26 October 2021, *Yuan 16439* (IFP 019951; paratype).

## ﻿Discussion

In this study, three new species of *Botryobasidium* collected from Southwestern China are described based on morphological characteristics and phylogenetic analyses combining ITS and LSU sequences. The molecular phylogenetic analyses showed moderate to high support in the deeper nodes and at the species level which is consistent with the previous study (Cao et al. 2021; Zhou et al. 2024).

The phylogenetic trees show that *B.acanthosporum* is closely linked to *B.incanum*, *B.isabellinum* and *B.vagum* (Figs 1, 2). *B.acanthosporum* resembles *B.incanum* and *B.vagum* in having pellicular and greyish basidiomes. However, the new species is unique by having spine-ornamented basidiospores. *B.acanthosporum* is similar to *B.isabellinum* in having yellowish basidiomes and spine-ornamented basidiospores, but *B.isabellinum* differs from the new species by having narrower subhymenial hyphae (6–8 μm), absence of cystidia, longer basidia (15–25 × 8–10 μm) and smaller globose basidiospores (7–10 μm) (Bernicchia and Gorjón 2010). In morphology, *B.bondarcevii* resembles *B.acanthosporum* in having pellicular and slightly yellow to dark yellow basidiomes, and spine-ornamented basidiospores, but *B.bondarcevii* can be distinguished by having bigger basidia (18–23 × 9–11 μm vs. 14.5–20 × 8–10 μm), ellipsoid basidiospores (Xiong et al. 2007).

In the phylogenetic trees (Figs 1, 2), *B.leptocystidiatum* grouped together with *B.subcoronatum*. Morphologically, they share similar characteristics in having thin and whitish to pale yellow basidiomes, clamped generative hyphae with frequently vertical branches, and subnavicular basidiospores. Nevertheless, *B.subcoronatum* differs from *B.leptocystidiatum* by the absence of cystidia, longer basidia and slightly narrower basidiospores (6–8 × 2.5–3 µm vs. 6.5–7.8 × 2.9–3.7 µm). Moreover, *B.leptocystidiatum* and *B.sassofratinoense* are similar in having greyish-white to yellow basidiomes and clamped hyphae. But *B.sassofratinoense* can be differentiated by having wider subhymenial hyphae (5–8 μm vs. 4–7 μm) and subicular hyphae (10–12 μm vs. 7–10 μm), shorter cystidia (28–45 × 5–7 μm vs. 21.5–77 × 4–7.5 μm), longer basidia (18–25 × 6–8.5 μm vs. 10.5–15 × 7–8 μm) and slightly bigger basidiospores (6.5–7.8 × 2.9–3.7 μm vs. 7–8.5 × 3.5–4.5 μm) (Bernicchia and Gorjón 2010).

*Botryobasidiumsubovalibasidium* has an adjacent phylogenetic relationship with *B.aureum*, *B.botryosum* and *B.candicans* in the phylogenetic trees (Figs 1, 2). In morphology, they exhibit some similarities in having whitish to yellowish basidiomes and absence of cystidia (Breitenbach and Kränzlin 1986; Bernicchia and Gorjón 2010). However, *B.aureum* is distinguished from the new species by having narrower subhymenial hyphae (4–6 µm) and subicular hyphae (5–10 µm) and smaller basidia (12–18 × 6–8 µm). *Botryobasidiumcandicans* differs from the new species by having narrower subhymenium and subicular hyphae, slightly smaller basidiospores (6–8 × 3–4 µm vs. 7–9.8 × 3.7–5.0 µm), and smaller chlamydospores (15–17 × 9–12 µm vs. 17–21 × 10–11 µm) (Bernicchia and Gorjón 2010). *Botryobasidiumbotryosum* can be separated from the new species by larger basidia, bigger basidiospores (8.5–11 × 4.4–5.5 μm vs. 7–9.8 × 3.7–5.0 µm), and absence of conidiospores (Jülich 1978). Moreover, *B.subovalibasidium* and *B.danicum* are similar in having greyish to yellowish basidiomes, absence of cystidia, and basidia with 4–6 sterigmata. However, *B.danicum* is distinct from the new species by longer basidiospores (12–14 × 3.0–5.0 µm vs. 7–9.8 × 3.7–5.0 µm), bigger basidia (15–20 × 8–12 µm vs. 14–18 × 9–10 µm) and absence of anamorphic spores (Bernicchia and Gorjón 2010).

### ﻿Key to known 18 species of *Botryobasidium* in China

**Table:** 

1	Basidiospores with spines	**2**
–	Basidiospores smooth	**4**
2	Basidiospores ellipsoid, 7–9 × 5–6.3 µm	***B.bondarcevii***
–	Basidiospores globose	**3**
3	Basidiospores 7–10 µm, spines up to 1–3 µm, basidia with 4 sterigmata	***B.isabellinum***
–	Basidiospores 8–10 µm, spines up to 4 µm, basidia with 2 sterigmata	***B.acanthosporum***
4	Conidia absent	**5**
–	Conidia present	**15**
5	Hyphae with clamps at least in a part of basidiome	**6**
–	Hyphae without clamps	**9**
6	Clamps present on all septa	**7**
–	Both clamps and simple septa present	**8**
7	Basidiospores navicular, 6–7 × 2.5–3 µm; cystidia absent	***B.subcoronatum***
–	Basidiospores subnavicular to navicular, 6.5–7.8 × 2.9–3.7 µm; cystidia present	***B.leptocystidiatum***
8	Clamps often present in subiculum and subhymenium	***B.angustisporum***
–	Clamps often absent in subiculum	***B.intertextum***
9	Basidiospores navicular	**10**
–	Basidiospores not navicular	**12**
10	Basidiospores 7–8 × 3–3.5 µm	***B.coniferarum***
–	Basidiospores more than 8 µm long	**11**
11	Basidiospores 9–10 × 3.5–5 µm; basidia cylindrical, 9–16 × 7–9 µm	***B.subbotryosum***
–	Basidiospores 8–12 × 4.5–6 µm; basidia clavate to subcylindrical, 20–25 × 8–12 µm	***B.vagum***
12	Basidiospores obliquely ovoid, apically obtuse	**13**
–	Basidiospores not ovoid	**14**
13	Basidiospores 7.5–12 × 3.5–5 µm	***B.obtusisporum***
–	Basidiospores 5–8 × 2.5–3.5 µm	***B.pruinatum***
14	Basidiospores subglobose, 14–17.5 × 13–15.5 µm	***B.gossypirubiginosum***
–	Basidiospores ellipsoid, 6.5–8.5 × 3.5–5 µm	***B.incanum***
15	Conidia ellipsoid	**16**
–	Conidia not ellipsoid	**17**
16	Conidia 13–22 × 9–12 µm; basidia ellipsoid to obovate, 12–15 × 6–8 µm	***B.conspersum***
–	Conidia 17–21 × 10–11 µm; basidia subovoid to ovoid, 14–18 × 9–10 µm	***B.subovalibasidium***
17	Conidia subglobose to citriform, 15–20 × 8–10 µm	***B.candicans***
–	Conidia subglobose to globose, 11.5–14.5 × 9.5–10.5 µm	***B.yunnanense***

## Supplementary Material

XML Treatment for Botryobasidium acanthosporum

XML Treatment for Botryobasidium leptocystidiatum

XML Treatment for Botryobasidium subovalibasidium

## ﻿Additional information

### Author ORCIDs

Lin-Jiang Zhou https://orcid.org/0000-0003-2665-6959

Xue-Long Li https://orcid.org/0009-0006-3948-0234

Hai-Sheng Yuan https://orcid.org/0000-0001-7056-140X

### Data availability

All of the data that support the findings of this study are available in the main text.
